# Geroscience approaches to obesity

**DOI:** 10.1093/geroni/igab046.3023

**Published:** 2021-12-17

**Authors:** Alessandro Bitto, Matt Kaeberlein

**Affiliations:** University of Washington, Seattle, Washington, United States

## Abstract

Besides aging, obesity is the greatest risk factor for numerous chronic pathologies, including metabolic syndrome, type 2 diabetes, cardiovascular disease, hypertension, and cancer. Preventing and treating obesity would greatly reduce healthcare costs and the impact of the aging process, with estimated savings up to $145,000,000,000. Pharmacological interventions identified by geroscience may prove effective against diet-induced obesity. To test this hypothesis, we fed a 66% kcal/fat diet to nine-month-old C57Bl6/N mice for 6 weeks and treated them with either rapamycin, acarbose, or a combination thereof. Rapamycin, and to a lesser extent acarbose, prevented weight gain and fat accumulation in these mice. We detected increased expression of the Liver Activating Protein (LAP) isoform of the transcription factor CCAT/Enhancer Binding Protein β (C/EBPβ) in the liver of mice treated with rapamycin. C/EBPβ-LAP mediates some of the effects of caloric restriction on nutrient metabolism and increases lifespan in a mouse transgenic model. We tested whether independent activation of C/EBPβ-LAP would recapitulate the effects of rapamycin by treating mice on a high-fat diet with adefovir dipivoxil, a reverse transcriptase inhibitor that can activate LAP in vitro independently of mTOR inhibition. Adefovir dipivoxil reduced weight and fat mass accumulation in mice over the course of 6 weeks. Mice treated with adefovir dipivoxil showed increased expression of genes involved in β-oxidation and lipids mobilization, and reduced activation of fatty acid biosynthesis and lipid storage pathways. Our results identify C/EBPβ-LAP as a potential new target to improve lipid homeostasis in both aging and obesity.

